# Combined detection of serum UL16-binding protein 2 and macrophage inhibitory cytokine-1 improves early diagnosis and prognostic prediction of pancreatic cancer

**DOI:** 10.3892/ol.2014.2429

**Published:** 2014-08-08

**Authors:** YU-FEN ZHOU, LING-XIAO XU, LI-YA HUANG, FANG GUO, FAN ZHANG, XIANG-YI HE, YAO-ZONG YUAN, WEI-YAN YAO

**Affiliations:** 1 Department of Gastroenterology, Ruijin Hospital Affiliated to Shanghai Jiaotong University School of Medicine, Shanghai, P.R. China; 2 Department of Gastroenterology, General Hospital of Ningxia Medical University, Yinchuan, Ningxia, P.R. China

**Keywords:** macrophage inhibitory cytokine-1, serum biomarker, pancreatic cancer, UL16-binding protein 2

## Abstract

Pancreatic cancer (PC) is the fourth leading cause of cancer-related mortality in the United States. There is no effective serum biomarker for the early diagnosis of PC at present. Although serum UL16-binding protein 2 (ULBP2) and macrophage inhibitory cytokine-1 (MIC-1) levels are reported to be elevated in PC patients, the diagnostic and prognostic value of ULBP2 and MIC-1 alone or in combination remains unknown. The aim of the present case-control study was to compare the diagnostic value of ULBP2, MIC-1 and carbohydrate antigen 19-9 (CA19-9) in 359 serum samples, consisting of 152 cases of PC, 20 cases of pre-pancreatic cancer, 91 cases of chronic pancreatitis (CP) and 96 normal controls (NC). All patients were followed up for a median of 2 years. It was found that the serum levels of ULBP2, MIC-1 and CA19-9 were significantly higher in the PC patients compared with those in the NC group. In distinguishing PC from the CP, the highest sensitivity and specificity were ULBP2 (0.878) and CA19-9 (0.816), respectively. The area under the receiver operating characteristic curve of ULBP2 was 0.923, which was the highest of the three biomarkers. MIC-1 was the optimal choice for the diagnosis of early-stage PC (area under the curve, 0.831). Overall, MIC-1 in combination with ULBP2 improved the diagnostic accuracy in differentiating PC from CP and NC. In addition, a higher level of MIC-1 was correlated with a poorer prognosis, as calculated by the Kaplan-Meier test (P=0.039). Patients with serum MIC-1 levels of ≥1,932 ng/ml had a median survival time of 15.62±2.44 months (mean ± standard deviation) vs. 18.66±2.43 months in patients with a lower level of MIC-1. Overall, combined detection of serum MIC-1 and ULBP2 improved the diagnostic accuracy in differentiating PC from CP and NC, and serum MIC-1 level alone was a predictor of survival in the patients with PC.

## Introduction

Pancreatic cancer (PC) is an aggressive malignancy with an overall five-year survival rate of only ~5% (1). The morbidity of PC is ranked tenth while mortality is ranked fourth among all cancers (2). The majority of PC patients have already lost the opportunity for radical resection at the time of diagnosis (1). The median survival time of patients with advanced PC is less than six months, while the five-year survival rate of resected minute pancreatic cancers (≤10 mm) can be >75% (3). Therefore, the early diagnosis of PC is of great significance to improve patient outcome.

Yachida *et al* (4) recorded a period of at least 15 years between the first mutation and the birth of the metastatic ability of PC. In addition, a retrospective study found that CT scans could diagnose asymptomatic PC six months prior to clinical diagnosis (5). However, no reliable non-invasive test is available at present for the early diagnosis of PC.

UL16-binding protein 2 (ULBP2) belongs to the ULBP family. The ULBPs are ligands for natural killer group 2, member D (NKG2D)/DNAX-activating protein of 10 kDa, an activating receptor expressed by natural killer (NK) cells (6). Macrophage inhibitory cytokine-1 (MIC-1) is a novel member of the TGF-β family (7). Knowing that ULBP2 and MIC-1 are highly expressed in PC, the aim of the present case-control study was to evaluate the diagnostic and prognostic value of ULBP2, MIC-1 and CA19-9 in PC.

## Material and methods

### Study design

A total of 359 subjects were enrolled in this study, consisting of 152 cases of PC, 20 cases of pre-pancreatic cancer (PPC; pancreatic intraepithelial neoplasia, intraductal papillary mucinous neoplasm and mucinous cystic neoplasm), 91 cases of chronic pancreatitis (CP) and 96 normal controls (NC). All the patients were admitted between 2009 and 2012 to the Department of Gastroenterology, Ruijin Hospital Affiliated to Shanghai Jiaotong University School of Medicine (Shanghai, China). The end-point of the follow-up period was April 2013, and a total of 100 follow-up records from the 152 PC patients were obtained. The study was approved by The Ethics Committee of Ruijin Hospital Affiliated to Shanghai Jiaotong University School of Medicine and all enrolled subjects signed informed consent documents.

The cytological or pathological evidence from the PC patients was obtained by endoscopic ultrasound, endoscopic retrograde cholangiopancreatography or surgery. PPC was diagnosed by pathology (8). Serum samples of the PC and PPC groups were collected prior to treatment. According to the sixth edition of the standard proposed in 2002 by the American Joint Committee on Cancer (9), tumor-node-metastasis (TNM) staging was performed on PC patients based on the post-operative pathological and CT scan findings. CP was defined based on clinical manifestations, including abdominal pain and steatorrhea, CT scan findings of calcifications, irregular pancreatograms and CP pathological changes obtained by surgery or secretin stimulation test to exclude PC. Baseline demographic information for all groups is detailed in Table I.

### Measurement of ULBP2 and MIC-1

All serum samples were frozen at −80°C until use. The serum levels of ULBP2 and MIC-1 were measured quantitatively by sandwich ELISA according to the manufacturer’s instructions, using the DuoSet ELISA kit (R&D Systems, Minneapolis, MN, USA) for human ULBP2 and MIC-1, respectively. The standard curve was plotted according to the manufacturer’s instructions. The detectable range of ULBP2 was between 15.6 pg/ml and 1 ng/ml, and the detectable range of MIC-1 was between 62.5 pg/ml and 4 ng/ml. Each sample was placed into two wells and detected twice. If the result was beyond the detection range, the serum sample was re-determined following appropriate dilution. A microplate reader was used to analyze the result at 450 nm, and corrected at 570 nm. CA19-9 was determined according to the manufacturer’s instructions for the solid phase radioimmunoassay in the Laboratory of Biochemical Medicine in the Ruijin Hospital Affiliated to Shanghai Jiaotong School of Medicine.

### Statistical analysis

Data were analyzed using IBM SPSS statistic 19 (IBM, Armonk, NY, USA). The Wilcoxon rank sum test and bilateral test were used in pairwise comparison of CA19-9, ULBP2 and MIC-1 serum levels between the PC, PPC, CP and NC groups. The correlation between the serum ULBP2 and MIC-1 levels and the patient characteristics in the PC group was evaluated by Spearman’s correlation analysis. A logistic regression analysis was used to draw a receiver operating characteristic (ROC) curve. The area under the curve (AUC) was calculated to compare the performance of different serum biomarkers as a diagnostic test. Survival data were analyzed using the Kaplan-Meier method with a log-rank test for comparison of survival curves. P<0.05 was considered to indicate a statistically significant difference.

## Results

### Serum ULBP2, MIC- and CA19-9 levels in each group

The serum ULBP2, MIC-1 and CA19-9 levels, shown for each group in Table II, were significantly higher in the PC patients than in the NC group (P<0.0001). In addition, there was a significant difference in the serum ULBP2 and MIC-1 levels between the PPC and NC groups (P=0.001 and P=0.003, respectively), but there was no statistically significant difference in serum CA19-9 between the two groups (P=0.063). There was no significant correlation between the ULBP2 levels and age, gender, tumor location, T stage, N stage, M stage or TNM stage in the PC group, although serum MIC-1 was significantly correlated with T stage (P=0.014).

### Comparison of diagnostic performances of ULBP2, MIC-1 and CA19-9 alone or a combination of two or three of these markers for PC diagnosis

**Figure 1:**
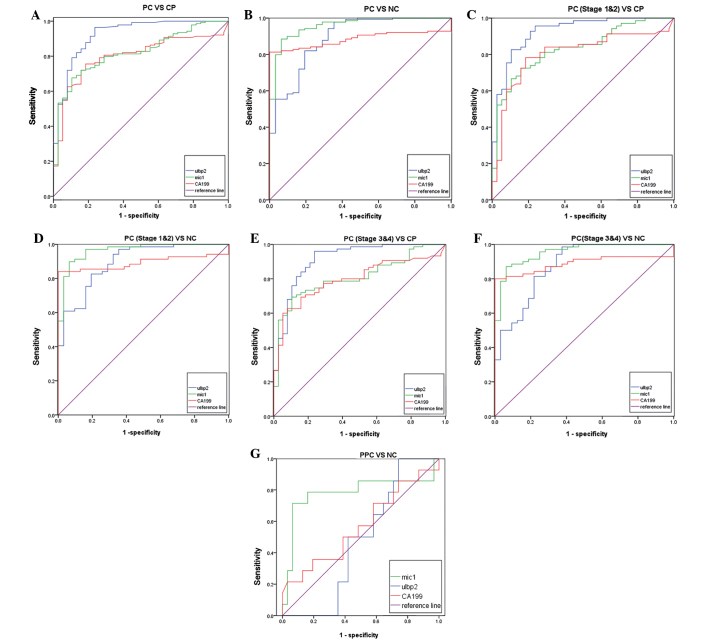
Diagnostic performance of ULBP2, MIC-1 and CA19-9 alone. (A) PC vs. CP. (B) PC vs. NC. (C) Stage 1-2 PC vs. CP. (D) Stage 1–2 PC vs. NC. (E) Stage 3–4 PC vs. CP. (F) Sstage 3–4 PC vs. NC. P-values of all receiver operating characteristic curves were <0.001. (G) PPC vs. NC; only serum MIC-1 could efficiently distinguish between PPC and NC (AUC, 0.781; 95% CI, 0.599–0.963; P=0.003). PC, pancreatic cancer; CP, chronic pancreatitis; PPC, pre-pancreatic cancer; NC, normal controls; CA19-9, carbohydrate antigen 19-9; ULBP2, UL16-binding protein 2; MIC-1, macrophage inhibitory cytokine-1; AUC, area under the curve.

Subsequently, the diagnostic performance of ULBP2, MIC-1 and CA19-9 alone was evaluated by plotting ROC curves and calculating the AUC. The optimal cut-off point was determined by ROC curve analysis. The sensitivity, specificity and AUC of different biomarkers in distinguishing PC from CP or NC are shown in Table III. The P-value of the AUC of the different groups was <0.001 in all groups. ULBP2 performed the best in the differential diagnosis of PC and CP, and also had the best sensitivity and specificity given the cut-off of ULBP2 at 86.12 pg/ml (Fig. 1A; Table III). Serum MIC-1 was effective in comparing PC and NC. When the cut-off values of CA19-9, ULBP2 and MIC-1 were set at 18.44 U/ml, 94.08 pg/ml and 642.83 pg/ml respectively, MIC-1 was found to have the highest sensitivity (89.9%), while CA19-9 had the highest specificity (96.8%) in distinguishing PC patients from NC (Fig. 1B; Table III).

Notably, MIC-1 was found to be the most effective marker [AUC, 0.964; 95% confidence interval (CI), 0.929–0.999; P<0.001] in distinguishing between stage 1–2 PC and NC. MIC-1 was also the most effective marker (AUC, 0.954; 95% CI, 0.916–0.992; P<0.001) in distinguishing between stage 3–4 PC patients and NC. ULBP2 was the most effective marker (AUC, 0.925; 95% CI, 0.873–0.978; P<0.001) in distinguishing between stage 1–2 PC and CP patients. ULBP2 was also the most effective marker in distinguishing between stage 3–4 PC and CP patients (Fig. 1C–F; Table IV). Only MIC-1 had diagnostic capability in distinguishing between PPC and NC (AUC, 0.781; 95% CI, 0.599–0.963; P=0.003). None of the biomarkers showed an efficient diagnostic performance in distinguishing between PPC and CP (Fig. 1G).

**Figure 2:**
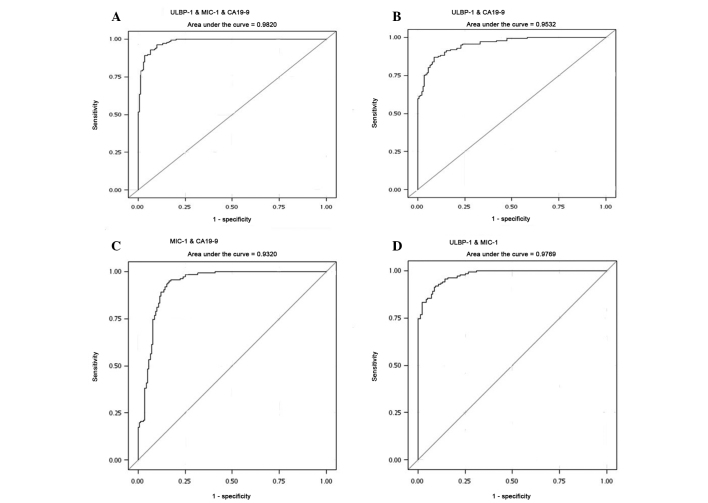
Combination of two or three markers for pancreatic cancer diagnosis compared with CA19-9 alone. (A) Combination of ULBP2, MIC-1 and CA19-9; AUC, 0.982. (B) Combination of ULBP2 and CA19-9; AUC, 0.953. (C) Combination of MIC-1 and CA19-9; AUC, 0.932. (D) Combination of ULBP2 and MIC-1; AUC, 0.977. P-values of all receiver operating characteristic curves were <0.001. CA19-9, carbohydrate antigen 19-9; ULBP2, UL16-binding protein 2; MIC-1, macrophage inhibitory cytokine-1; AUC, area under the curve.

A combination of any two or three of these biomarkers improved the diagnostic performance in distinguishing between PC and CP. Combination of the three biomarkers showed the largest AUC (0.982; P<0.001). Combination of any two of the three biomarkers showed that MIC-1 combined with ULBP2 produced the optimal diagnostic performance (AUC, 0.977; P<0.001; Fig. 2).

### Correlations between ULBP2, MIC-1 and PC survival

**Figure 3:**
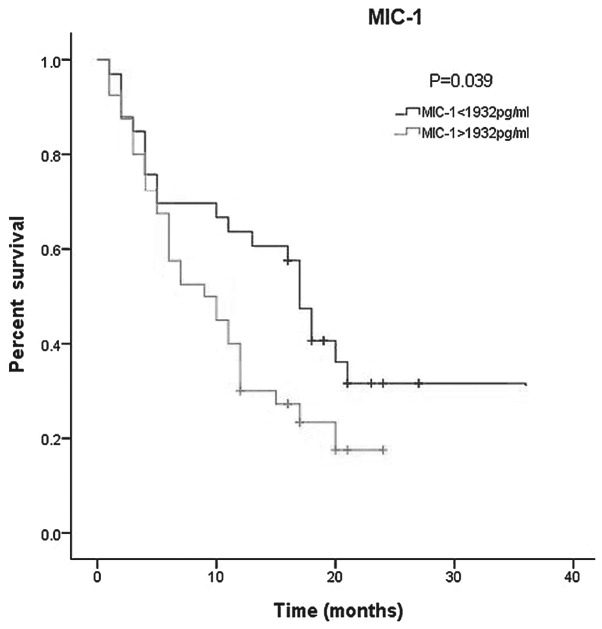
Overall survival of PC stratified by serum MIC-1. Median survival time was 18.66±2.43 months in PC patients with a serum MIC-1 level of ≤1,932 pg/ml (95% CI, 13.91–23.42) vs. 15.617±2.44 months in patients with a serum MIC-1 level of >1,932 pg/ml (95% CI, 10.83–20.41). P=0.039 and χ^2^=4.239. PC, pancreatic cancer; MIC-1, macrophage inhibitory cytokine-1; CI, confidence interval.

Survival data were analyzed using the Kaplan-Meier method with log-rank test for comparison. In this study, the median was set as the cut-off point. It was found that MIC-1 was correlated with the prognosis of PC. A higher serum level of MIC-1 indicated a worse prognosis. The median survival of PC patients with a serum MIC-1 level of ≤1,932 pg/ml was 18.66±2.43 months (95% CI, 13.91–23.42), while a higher MIC-1 level was associated with a shorter survival duration averaging 15.617±2.44 months (95% CI, 10.83–20.41) (Fig. 3). No correlation was found between the serum ULBP2 level and PC prognosis.

## Discussion

CA19-9 is currently the most commonly used serological marker in the diagnosis of PC, but its performance in early diagnosis is limited. Therefore, it is more often used in the follow-up of PC patients after surgery or chemotherapy (10–12). In addition, elevated serum CA19-9 levels have been reported in a number of gastrointestinal diseases, such as pancreatitis, hepatitis and biliary obstruction. Serum CA19-9 levels are particularly increased in patients with biliary obstruction (13). These findings compromise the specificity of CA19-9. Clearly, determining novel PC biomarkers to circumvent this drawback of CA19-9 has good prospects for development. In the present study, it was found that serum MIC-1 and ULBP2 improved the diagnostic accuracy in differentiating between PC, CP and NC, and that serum MIC-1 levels could be used to predict survival in PC patients.

ULBP2 and MIC-1 have been reported to be overexpressed in various types of cancer tissue and to be secreted by cancer cells, including PC cells (14–17). This is consistent with the findings of the present study showing that serum ULBP2 and MIC-1 levels were significantly higher in the PC patients than in the NC and CP patients. In addition, the study was the first to find that serum MIC-1 and ULBP2 expression was significantly elevated in the PPC patients compared with the NC (P=0.003), while there was no significant difference in CA19-9 between the two groups, suggesting that ULBP2 and MIC-1 could be used for the early diagnosis of PC.

The early diagnosis of PC remains a clinical challenge at present. Although numerous studies have demonstrated that multiple serum biomarkers, including peptidylglycine α-amidating monooxygenase 4, heat shock protein 27 and tumor-specific growth factor, are elevated in PC, the diagnostic performance of these biomarkers alone is not sufficient for clinical application, particularly due to their limited capacity for the early diagnosis of PC (18). The present study first confirmed that ULBP2 and MIC-1 alone outperformed CA19-9 in identifying PC, with high specificity and sensitivity. The combination of ULBP2, MIC-1 and CA19-9 enhanced the efficacy of the PC diagnosis. Subsequently, it was found that ULBP2 and MIC-1 performed better than CA19-9 in distinguishing early-stage, stage 1–2, PC. More significantly, by plotting ROC curves and calculating the AUC, it was found that only MIC-1 had the diagnostic capability for distinguishing between PPC and NC (AUC, 0.781; 95% CI, 0.599–0.963; P=0.003). Therefore, serum ULBP2 and MIC-1 are useful biomarkers for the diagnosis of PC, particularly in the diagnosis of early-stage PC.

In addition, this study also analyzed the correlation between ULBP2 and MIC-1, and PC prognosis. The result showed that serum MIC-1 was correlated with PC prognosis.

The molecular mechanism of ULBP2 and MIC-1 underlying the development of PC remains unclear. ULBP2 is one of the ligands for NKG2D. When the normal epithelium is transformed, the stress-induced ligands for NKG2D (human MIC and ULBP antigens) may be expressed on the cell surface (19). ULBP2 on the tumor cell surface can bind to NKG2D receptors on immune cells, such as NK cells and CD8^+^ T cells, ultimately inducing the innate immune response of killing and scavenging tumor cells (20). ULBP2 expression is regulated by tumor suppressors at the transcriptional and post-translational levels. For example, p53 functions as a direct transcriptional activator of ULBP2 and represses ULBP2 translation by upregulating microRNA (miR)-34a and miR-34b/c. The ultimate ULBP2 level is determined by the balance of the two regulatory mechanisms (21).

MIC-1 was first reported by Bootcov *et al* in 1997, and is recognized as a divergent member of the transforming growth factor-β (TGF-β) superfamily, which plays a complex role in several human diseases, including cancer (7). MIC-1 can serve as a potential diagnostic and prognostic biomarker for certain cancers (22). A number of studies have shown that MIC-1 can activate multiple signaling pathways, including the focal adhesion kinase signaling pathway, the extracellular signal regulated kinase 1/2 signaling pathway and the phosphatidylinositol 3′-kinase-Akt intracellular pathway, although the identity of the MIC-1 binding ligand is not clear (23–25). MIC-1 is the only known cytokine to regulate secreted p53, which is expressed strongly in the presence of active p53 (26,27). Although MIC-1 plays an antitumor role in the early stages of cancer, it promotes invasion and metastasis in advanced stages. Johnen *et al* reported that serum MIC-1 was closely associated with sustained weight loss in cancer patients (28).

In conclusion, the present study demonstrated that ULBP2 and MIC-1 could be used potentially as serum biomarkers in the diagnosis of PC, particularly when they are used in combination. In addition, serum MIC-1 could be used to predict the PC outcome. Further studies are required to clarify the molecular mechanisms of ULBP2 and MIC-1 underlying the development of PC.

## Figures and Tables

**Table I:** Demographics and clinicopathologic characteristics of the patients.

Characteristic	PC	CP	NC	PPC
No. of patients	152	91	96	20
Age				
Mean (SD), years	56 (13.5)	58 (15.0)	58 (7.6)	60 (11.3)
>60, n	88	58	53	6
≤60, n 64	33	43	14	
Gender, n				
Males	101	52	72	15
Females	51	39	24	5
Stage, n				
IA	5			
IB	12			
IIA	36			
IIB	20			
III	40			
IV	39			
CA19-9, U/ml^a^	1448.78±3707.04	38.23±138.96	7.69±4.89	10.44±7.40
CEA, ng/ml^a^	15.15±94.79	2.74±2.09	1.27±2.05	2.63±1.25

a Data are presented as the mean ± SD.

PC, pancreatic cancer; CP, chronic pancreatitis; PPC, pre-pancreatic cancer; NC, normal controls; CA19-9, carbohydrate antigen 19-9; CEA, carcinoembryonic antigen; SD, standard deviation.

**Table II:** Comparison of serum ULBP2, MIC-1 and CA19-9 within groups, mean ± standard deviation.

Biomarkers	PC (n=152)	CP (n=91)	PPC (n=20)	NC (n=96)
CA19-9, U/ml	1448.78±3707.04	38.23±138.96	10.44±7.40	7.69±4.89
ULBP2, pg/ml	219.89±182.48	68.33±36.78	76.51±40.9	62.62±11.37
MIC-1, pg/ml	3521.34±3903.38	959.61±878.98	973.59±588.89	427.61±316.95

PC, pancreatic cancer; CP, chronic pancreatitis; PPC, pre-pancreatic cancer; NC, normal controls; CA19-9, carbohydrate antigen 19-9; ULBP2, UL16-binding protein 2; MIC-1, macrophage inhibitory cytokine-1.

**Table III:** Sensitivity, specificity and AUC of different biomarkers in distinguishing between PC, CP and NC.

Markers (optimal cut-off)	AUC	95% CI	P-value	Sensitivity	Specificity
PC vs. CP					
MIC-1 (983.20 pg/ml)	0.820	0.753–0.887	<0.001	0.806	0.632
ULBP2 (86.12 pg/ml)	0.923	0.871–0.975	<0.001	0.878	0.816
CA19-9 (36.2 U/ml)	0.799	0.728–0.870	<0.001	0.763	0.816
PC vs. NC					
MIC-1 (642.83 pg/ml)	0.958	0.924–0.992	<0.001	0.899	0.903
ULBP2 (94.08 pg/ml)	0.889	0.824–0.955	<0.001	0.820	0.806
CA19-9 (18.44 U/ml)	0.883	0.833–0.932	<0.001	0.820	0.968

PC, pancreatic cancer; CP, chronic pancreatitis; PPC, pre-pancreatic cancer; NC, normal controls; CA19-9, carbohydrate antigen 19-9; ULBP2, UL16-binding protein 2; MIC-1, macrophage inhibitory cytokine-1; AUC, area under the curve; CI, confidence interval.

**Table IV:** AUC of different biomarkers in distinguishing stage1–2 PC or stage 3–4 PC from CP or NC.

PC stage	CP AUC	95% CI	NC AUC	95% CI
Stage 1–2 PC				
MIC-1	0.831	0.754–0.908	0.964	0.929–0.999
ULBP2	0.925	0.873–0.978	0.896	0.831–0.961
CA19-9	0.802	0.714–0.891	0.892	0.826–0.959
Stage 3–4 PC				
MIC-1	0.809	0.730–0.889	0.954	0.916–0.992
ULBP2	0.913	0.853–0.972	0.875	0.800–0.950
CA19-9	0.795	0.713–0.877	0.888	0.822–0.955

PC, pancreatic cancer; CP, chronic pancreatitis; PPC, pre-pancreatic cancer; NC, normal controls; CA19-9, carbohydrate antigen 19-9; ULBP2, UL16-binding protein 2; MIC-1, macrophage inhibitory cytokine-1; AUC, area under the curve; CI, confidence interval.
